# Genetic variation between long-lived versus short-lived bats illuminates the molecular signatures of longevity

**DOI:** 10.18632/aging.103725

**Published:** 2020-08-31

**Authors:** Zixia Huang, Conor V. Whelan, Dina Dechmann, Emma C. Teeling

**Affiliations:** 1 School of Biology and Environmental Science, University College Dublin, Belfield, Dublin, Ireland; 2 Department of Migration, Max Planck Institute of Animal Behavior, Radolfzell, Germany; 3 Department of Biology, University of Konstanz, Konstanz, Germany; 4 Smithsonian Tropical Research Institute, Panama

**Keywords:** bats, longevity, comparative genomics, transcriptomics, autophagy

## Abstract

Bats are the longest-lived mammals given their body size with majority of species exhibiting exceptional longevity. However, there are some short-lived species that do not exhibit extended lifespans. Here we conducted a comparative genomic and transcriptomic study on long-lived *Myotis myotis* (maximum lifespan = 37.1 years) and short-lived *Molossus molossus* (maximum lifespan = 5.6 years) to ascertain the genetic difference underlying their divergent longevities. Genome-wide selection tests on 12,467 single-copy genes between *M. myotis* and *M. molossus* revealed only three genes (*CCDC175*, *FATE1* and *MLKL*) that exhibited significant positive selection. Although 97.96% of 12,467 genes underwent purifying selection, we observed a significant heterogeneity in their expression patterns. Using a linear mixed model, we obtained expression of 2,086 genes that may truly represent the genetic difference between *M. myotis* and *M. molossus*. Expression analysis indicated that long-lived *M. myotis* exhibited a transcriptomic profile of enhanced DNA repair and autophagy pathways, compared to *M. molossus*. Further investigation of the longevity-associated genes suggested that long-lived *M. myotis* have naturally evolved a diminished anti-longevity transcriptomic profile. Together with observations from other long-lived species, our results suggest that heightened DNA repair and autophagy activity may represent a universal mechanism to achieve longevity in long-lived mammals.

## INTRODUCTION

Natural selection has shaped a large variation of lifespan across mammals, with maximum lifespan ranging from a few months (e.g. short-lived shrews) to 211 years (e.g. bowhead whale) [1]. Although the bowhead whale is exceptionally long-lived, its lifespan is arguably not as extreme as that of a 30 years old naked mole-rat given their body sizes, as maximum lifespan (MLS) exhibits a positive correlation with body size within mammals [2, 3]. Thus, lifespan comparison across mammals requires body size correction. To resolve this, the longevity quotient (LQ) was introduced, which is defined as the ratio of observed lifespan to predicted lifespan for a non-flying mammal of the same body size [2, 3]. Using this approach bats are the longevity extremists, with some species living up to ten times longer than expected given their body size [2]. The Brandt’s bat (*Myotis brandtii*) holds the record for longevity [4], with a maximum lifespan of >40 years, living 8~10 times longer than expected given body size (~7 grams) [2, 4, 5]. This renders bats as one of the most ideal taxa to explore the molecular basis of extraordinary longevity in mammals.

Although the majority of bat species are long-lived, especially within the *Myotis* genus, there are a few short-lived exceptions, such as the velvety free-tailed bat (*Molossus molossus*) and the evening bat (*Nycticeius humeralis*), living as long as would be expected given their body size [5, 6]. A recent study has suggested that the ancestral bat lived up to 2.6 times longer than expected given body size, indicating that the extreme longevity observed in the longest-lived bat genera may have evolved multiple times [7]. This also suggests that short-lived bat species may have lost their longevity adaptations. Therefore, this wide range of lifespans observed in bats enables us to utilize comparative evolutionary approaches to search for genetic differences within closely-related long- and short-lived bat species. In contrast to comparative studies on phylogenetically-distant species (e.g. bats versus mice), this comparison could minimize the ‘noise’ resulting from heterogenous physiology, and may reveal key anti-aging molecular adaptations that have evolved in long-lived bats, or were lost in their short-lived counterparts.

Genome-wide comparative analyses have been carried out on a few long- and short-lived species in primates [8], rodents [9, 10], whales [11] and bats [12]. These studies revealed a few genes that showed convergent amino acid mutations, or exhibited positive selection, or were differentially expressed in long-lived species compared with their short-lived counterparts. Although there is little commonality across the gene candidates that are associated with longevity revealed by these studies, they are mainly enriched in DNA repair and maintenance, autophagy, homeostasis, and nutrient sensing pathways [13]. In bats, our previous longitudinal studies showed that long-lived *M. myotis* bats maintained their transcriptomic profiles [14] and telomere length [5], and did not exhibit an increased level of mitochondrial damage with advancing age [15], all of which likely contribute to their extraordinary longevity. However, a parallel comparison between long- and short-lived bats is lacking.

In this study we performed a comparative genomic and transcriptomic analysis between long-lived *Myotis myotis* (MLS = 37.1 years; LQ = 5.71) and short-lived *Molossus molossus* (MLS = 5.6 years; LQ = 0.99) [6] to ascertain the molecular signatures associated with longevity in bats. Based on the genome-wide alignments of single-copy orthologous genes between these two species, we detected and further investigated the genes that were fast-evolving and showed significant positive selection. We also deep sequenced blood transcriptomes from eight adult individuals for each species, and explored the genes and pathways that were differentially expressed. To ascertain if long-lived bats have evolved a transcriptomic signature of longevity, we further investigated the expression of ‘pro’- and ‘anti’-longevity genes in the blood transcriptomes of *M. myotis* and *M. molossus*. Although the majority of genes underwent purifying selection, we observed a significant transcriptional alteration between these two species. Among 2,086 genes that exhibited large interspecific expression variation, the genes that showed higher expression in long-lived *M. myotis* were mainly enriched in DNA repair and autophagy. Further pathway analysis suggested that six biological processes, including autophagy, were differentially expressed between *M. myotis* and *M. molossus*. We also show that *M. myotis* had significantly lower expression levels of anti-longevity genes, suggestive of a transcriptomic signature of longevity naturally evolved in long-lived bats. Together with the previous findings in other long-lived mammals, our study implies that enhanced DNA repair and autophagy activity may represent a universal mechanism to achieve longevity in long-lived mammals.

## RESULTS

### The majority of genes undergo purifying selection

**Figure 1:**
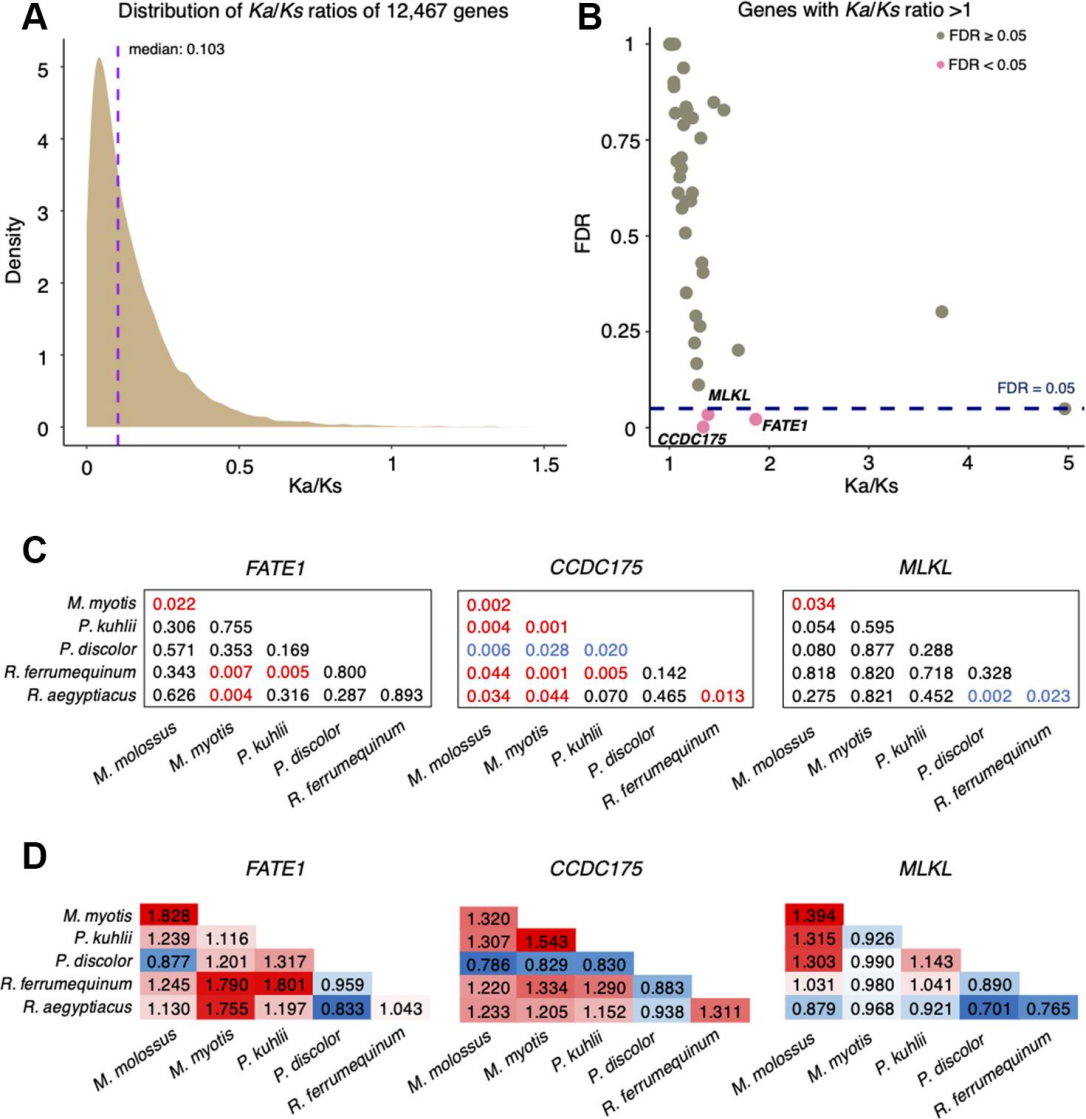
**Analysis of *Ka/Ks* substitution rates of 12,467 single-copy genes between *M. myotis* and *M. molossus*.** (**A**) Distribution of *Ka*/*Ks* ratios of 12,467 single-copy genes. To better visualize the distribution, six genes with *Ka*/*Ks* > 1.5 were not included in this plot. (**B**) Genes with *Ka*/*Ks* > 1. Three genes highlighted in red show significant positive selection (*Ka*/*Ks* > 1; FDR < 0.05 Fisher’s exact test). (**C**) Significance (FDR) of *Ka*/*Ks* ratios of *FATE1*, *CCDC175* and *MLKL* between 6 bat species through pairwise comparisons. The red values indicate significant positive selection while the blue values indicate significant purifying selection. The black values indicate no selection. (**D**) *Ka*/*Ks* ratios of *FATE1*, *CCDC175* and *MLKL* between 6 bat species through pairwise comparisons. The red values indicate *Ka*/*Ks* ratios > 1 while the blue values indicate *Ka*/*Ks* ratios < 1.

To evaluate the natural selection acting on protein-coding genes between *M*. *myotis* and *M*. *molossus*, we calculated the ratio of *Ka* and *Ks* substitution rates for each pair of orthologous genes. We observed that most of the genes (97.96%) were under purifying selection (*Ka*/*Ks* ratio < 1, FDR < 0.05), with the median of *Ka*/*Ks* ratios equal to 0.103 (Figure 1A). In total, 48 genes had the ratios of *Ka*/*Ks* > 1, only three of which (*FATE1*, *MLKL* and *CCDC175*) exhibited significant positive selection (Fisher’s exact test, FDR < 0.05, Figure 1B). To determine if positive selection on these genes was the consequence of species-specific selective pressures, we conducted pairwise analyses of orthologous genes across 6 bat species (See Methods). We found that each comparison resulted in a unique set of positively selected genes (PSGs) (Supplementary Table 1). Interestingly, positive selection on *FATE1* and *CCDC175* was also observed between *M. molossus*, *M. myotis* and other bat species (Supplementary Table 1). Although *MLKL* was solely seen under significant positive selection between *M. myotis* and *M. molossus*, this gene also showed high *Ka*/*Ks* ratios between all possible comparisons of bat species (Figure 1C and 1D).

### Transcriptomic profiles exhibit a substantial difference in long-lived and short-lived bats

**Figure 2:**
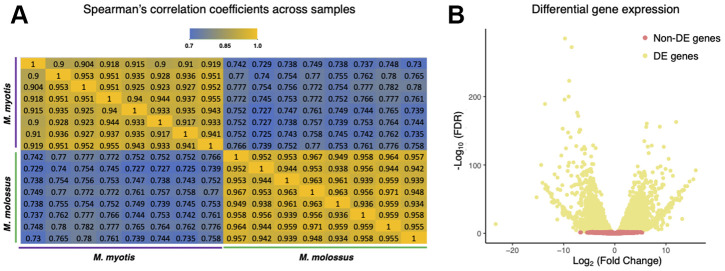
**Comparisons of *M. myotis* and *M. molossus* blood transcriptomes.** (**A**) Spearman’s correlation coefficients between *M. myotis* and *M. molossus* blood transcriptomes based on expression levels of 10,635 single-copy genes. We excluded 1,832 genes that were neither expressed in *M. myotis* nor *M. molossus*. (**B**) Differential gene expression analysis between *M. myotis* and *M. molossus* blood transcriptomes. Genes with FDR < 0.05 were considered differentially expressed genes (DEGs).

To ascertain the differences on the transcriptional level, we further sequenced and compared their blood transcriptomes (n = 16) using Illumina RNA-Seq. Out of 12,467 orthologs, we excluded 1,832 genes that were not expressed in either *M*. *molossus* or *M*. *myotis* and retained 10,635 genes for downstream analyses. The correlation analysis revealed a considerable difference in global transcriptomic profiles between *M*. *molossus* and *M*. *myotis* (Figure 2A, See Methods). The samples showed statistically higher intraspecific Spearman’s correlation coefficients (ρ = 0.957) in contrast to the interspecific counterparts (ρ = 0.766) (*P* = 2.2×10^-16^, Wilcoxon signed-rank test). In addition, 4,846 (45.57%) genes were differentially expressed (FDR < 0.05). 2,162 (20.33%) genes showed up-regulation in *M*. *myotis* compared to *M*. *molossus* while 2,684 (25.24%) genes were down-regulated (Figure 2B). Interestingly, there was no significant difference in the distribution of *Ka*/*Ks* ratios between differentially and non-differentially expressed genes (*P* = 0.162, Kolmogorov-Smirnov test). Next, we tested if the *Ka*/*Ks* ratios of 10,635 orthologs had an association with their differential expression patterns in *M. molossus* and *M. myotis*. We found that there was no significant association between the genes differentially expressed and their *Ka*/*Ks* ratios (*P* = 0.352, *χ*^2^ test, See Methods).

### DNA repair and autophagy are enriched by the genes that have genetically higher expression in long-lived bats

**Figure 3:**
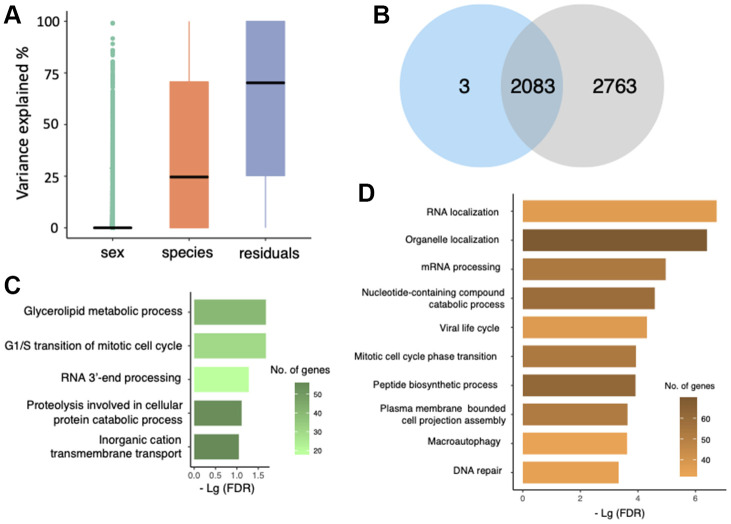
**Gene expression variation analysis.** (**A**) Evaluation of gene expression variance using a linear mixed model. Residual variance represents the contribution from uncharacterized variables. (**B**) Overlap of differentially expressed genes (blue) and the genes with at least 80% of expression variation resulted from ‘species’ (grey). (**C**) GO terms that were enriched by 1,060 genes that had higher expression in *M. molossus*. (**D**) GO terms that were enriched by 1,026 genes that had higher expression in *M. myotis*.

For each of 10,635 genes, we calculated the proportion of interspecific expression variation using a linear mixed model (See Methods). On average, 24.9% of gene expression variance was explained by ‘species’ while ‘sex’ only explained a small proportion of variation (Figure 3A). To investigate the gene expression that genetically differed in *M. myotis* and *M. molossus*, we focused on 2,086 genes with at least 80% of their expression variance resulted from ‘species’ which represented interspecific variation (See Methods). As was expected, 2,083 of these genes (99.86%) were also detected as differentially expressed genes (DEGs) (Figure 3B).

In total, 1,060 genes had higher expression levels in *M. molossus* while 1,026 genes in *M. myotis*. GO terms that were enriched for the genes with higher expression in short-lived bats include glycerolipid metabolic process, G1/S transition of mitotic cell cycle and RNA 3’-end processing (Figure 3C). In contrast, the genes that had higher expression in long-lived bats were enriched in a variety of biological processes such as RNA localization, viral life cycle and peptide biosynthetic process (Figure 3D). Interestingly, we observed that DNA repair and macroautophagy were also enriched for the genes that exhibited higher expression in long-lived bats (Figure 3D).

### Pathway analysis reveals up-regulated autophagy pathways in long-lived bats

**Figure 4:**
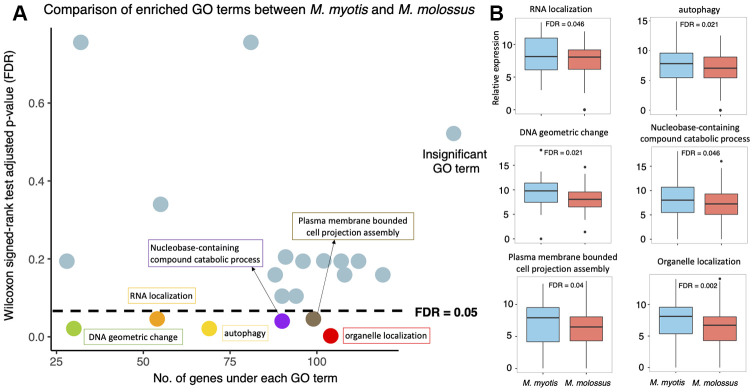
**Expression analysis of the 20 GO terms between *M. myotis* and *M. molossus*.** (**A**) Differential expression analysis of GO terms enriched by 2,086 genes that showed >80% interspecific expression variation. Differentially expressed GO terms were determined by comparing gene expression under each GO term using Wilcoxon signed-rank test (paired mode; one-tailed test). GO terms with FDR < 0.05 were considered differentially expressed. (**B**) Distribution of gene expression under each of 6 differentially expressed GO terms between *M. myotis* and *M. molossus*.

To identify the biological processes that were differentially expressed between short-lived and long-lived bats, we investigated the 20 GO terms that were enriched by these 2,086 genes. We compared expression levels of all the genes under each enriched GO term between *M. myotis* and *M. molossus* using Wilcoxon signed-rank test (paired mode, one-tailed test) (See Methods). Out of these 20 terms, the genes under 6 terms had significantly higher expression in long-lived bats than in short-lived bats (FDR < 0.05) (Figure 4A). These GO terms include DNA geometric change (GO:0032392), RNA localization (GO:0006403), autophagy (GO:0006914), nucleobase-containing compound catabolic process (GO:0034655), plasma membrane bounded cell projection assembly (GO:0120031), and organelle localization (GO:0051640) (Figure 4B). In contrast, none of these 20 GO terms exhibited significantly higher expression in *M. molossus* than in *M. myotis*, respectively (Wilcoxon signed-rank test, paired mode, one-tailed, FDR < 0.05). The genes under each of these 20 enriched GO terms are available in Supplementary Table 2.

### Long-lived bats have evolved a transcriptomic signature of longevity

**Figure 5:**
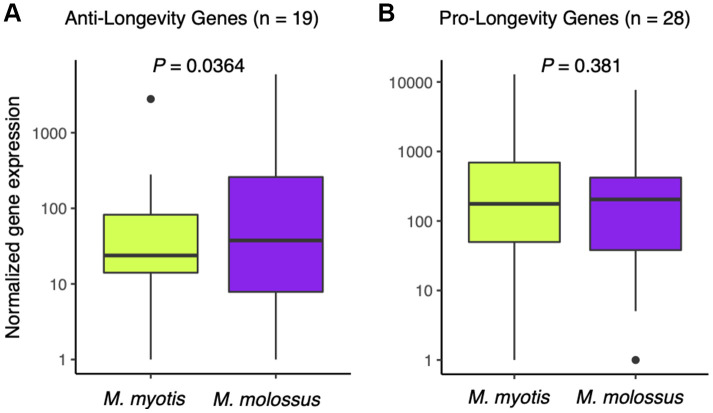
**Expression analysis of anti- and pro-longevity genes between *M. myotis* and *M. molossus*.** (**A**) Comparison of anti-longevity gene expression (n = 19) between *M. myotis* and *M. molossus* using Wilcoxon signed-rank test (paired mode, one-tailed test). (**B**) Comparison of pro-longevity gene expression (n = 28) between *M. myotis* and *M. molossus* using Wilcoxon signed-rank test (paired mode, one-tailed test).

To ascertain whether long-lived bats have naturally evolved a transcriptomic signature of longevity, we compiled a list of anti-longevity and pro-longevity genes and compared their expression in the *M. myotis* and *M. molossus* blood transcriptomes. We observed that there was no significant difference in the expression of the pro-longevity genes (n = 28) in long-lived and short-lived bats (*P* = 0.381, Wilcoxon signed-rank test, paired mode, one-tailed, Figure 5A), while the anti-longevity genes (n=19) had significantly lower expression in long-lived bats (*P* = 0.0364, Wilcoxon signed-rank test, paired mode, one-tailed, Figure 5B). To assess its significance, we randomly subsampled 19 genes out of 2,086 genes for 1,000 times, and performed Wilcoxon signed-rank tests as stated above and analysed the distribution of the *P*-values. We noticed that *P* = 0.0364 fell outside the range of 95% highest density interval (lower: 0.0844, upper: 0.998), suggesting that the significance of the test did not result from random chance.

## DISCUSSION

Identifying positive selection is a powerful approach to detect genomic adaptations that may contribute to species-specific phenotypes. In this study, we aimed to identify signatures of positive selection within a short-lived bat species (*Molossus molossus*) and a longer-lived bat species (*Myotis myotis*) to reveal genomic adaptations that correlate with longevity. Similar to previous studies examining positive selection in bat lineages [16, 17], the majority of genes investigated in this study were under purifying selection, which is not surprising given the evolutionary constraint on protein-coding genes. However, signs of significant positive selection were detected in 3 genes (*FATE1*, *MLKL* and *CCDC175*) between *M. molossus* and *M. myotis*, two of which (*FATE1* and *CCDC175*) were also under significant positive selection among other bat species (Supplementary Table 1). This suggests that *FATE1* and *CCDC175* evolved fast along all bat lineages examined and their positive selection might not be associated with longevity. We noticed that *MLKL* was uniquely seen under significant positive selection between *M. molossus* and *M. myotis* (Figure 1C). *MLKL* is a key signaling molecule in the necroptosis pathway, a programmed cell death process [18], and deregulation of this gene has been reported to promote necroptosis-induced inflammation in aged mice [19]. Interestingly, a recent study investigating several long- and short-lived rodents revealed a different set of PSGs, which were enriched in cellular homeostasis and mTOR pathway [10]. The disparity of PSGs may result from different taxa and methods used in these two studies or suggest that the longevity mechanisms evolved in long-lived mammals are species-specific. In our study, we only examined two bat lineages that have extremely divergent lifespans. As more and more high-quality bat genomes will become available [20], a more powerful and comprehensive investigation, such as applying branch-site tests for positive selection to multiple long- and short-lived branches, is required.

Although we did not observe strong signals of positive selection on these protein-coding genes, we did notice a significant difference in their expression between *M. myotis* and *M. molossus*. Interestingly, we showed that differential or non-differential expression of these genes had no association with the selective pressures acting on them (*P* = 0.352, *χ*^2^ test). Only one of the three PSGs (*FATE1*) found in our study was differentially expressed. Therefore, we hypothesize that differential gene expression is likely due to post-transcriptional regulation of non-coding genes rather than natural selection on these protein-coding genes [21].

Differential expression analysis measures the difference in mean expression of genes between these two species but fails to uncover the intraspecific gene expression variation [22]. Transcriptomes are subjected to change due to instant alterations of physiological status or environmental context. As the biometrics of *M. myotis* and *M. molossus* individuals used in this study, such as age, disease status and environmental circumstances, are unknown, a large proportion of intraspecific transcriptional variation is expected. One purpose of this study is to identify gene expression that genetically differs in these two species; hence we need to explore the genes which have high interspecific but low intraspecific expression variation. To resolve this, we employed a linear mixed model to quantify gene expression variation (See Methods). We focused on 2,086 genes with at least 80% of their overall expression variance resulted from interspecific difference, and indeed, a huge overlap between these genes and DEGs was revealed (Figure 3B). We excluded 2,763 DEGs with large proportions of intraspecific and other unknown variation from further analyses, as their differential expression might not reflect genetic difference between these two species.

Among these 2,086 genes, we noticed that the genes having higher expression in long-lived *M. myotis* were enriched in DNA repair and autophagy. For example, *WRN* and *XPC* were expressed 6.7-fold and 4.8-fold higher in *M. myotis* compared to *M. molossus*, respectively. *WRN* has been shown to regulate the repair of DNA double strand breaks (DSBs) and may play a role in telomere maintenance [23], while *XPC* may act as a general sensor of damaged DNA and involve in base excision repair (BER) [24]. Genomic stability is under constant challenge by intrinsic and extrinsic agents [25]. In most mammals, the capability of genomic maintenance attenuates with advancing age, leading to accumulation of DNA damage and thus, age-related phenotypes [26, 27]. However, together with our previous findings in long-lived *M. myotis* bats [14], multiple lines of evidence have revealed up-regulation of genes directly involved in DNA damage signalling and repair in a few long-lived mammals during aging, such as human [28], naked mole-rat [28] and bowhead whale [11]. In addition, autophagy is a critical recycling pathway which maintains cellular metabolism and energy homeostasis [29, 30]. We found that *UVRAG*, which was identified as a tumor suppressor, was up-regulated 13.7-fold in long-lived *M. myotis* compared to *M. molossus*. *UVRAG* induces autophagosome formation and maturation, and its overexpression promotes autophagy and suppress tumor cell growth [31]. Other autophagy-related genes (e.g. *ATG3*, *ATG4A*, *ATG4C*) also exhibited significantly higher expression in *M. myotis*. Increased autophagy activity may allow long-lived *M. myotis* to better counteract the age-related accumulation of damaged proteins and organelles, thus to improve the metabolic fitness of cells. Consistent with our finding, recent studies on rodents [32] and whales [11] show that autophagy is significantly enhanced in long-lived species relative to their short-lived counterparts. These results imply that enhanced DNA repair and autophagy activity may represent a universal mechanism to achieve longevity in long-lived species, independent of their phylogenetic distance. Interesting, we also found that the genes enriched in ‘Viral life cycle’ had significantly higher expression in *M. myotis* (Figure 3D). This may be due to the possibility that the *M. myotis* individuals, sampled from the colonies in France, carried a higher viral load or could represent naturally evolved anti-viral mechanism in long-lived bats. This may result in activation of the genes that control viral replication in *M. myotis*. Coupled with some other enriched GO terms (e.g. RNA localization, glycerolipid metabolic process and mitotic cell cycle phase transition), its roles in the aging process in bats will need further exploration.

Focusing on these 2,086 genes, we further explored the biological processes and expression of all the genes that were enriched under each GO term (See Methods). We found that, compared to *M. molossus*, the genes under 6 enriched GO categories had significantly higher expression in *M. myotis*, respectively (Figure 4A and 4B, Wilcoxon signed-rank test, FDR-adjusted *P* < 0.05). Apart from autophagy which has a crucial role in the regulation of lifespan, the other 5 terms have not been clearly linked with aging yet. These 5 differentially expressed GO categories may indicate the general transcriptomic differences between these two species.

Next, we investigated a list of pro-longevity and anti-longevity genes [33] to ascertain if long-lived bats have naturally evolved a transcriptomic profile of longevity. These genes are mainly involved in DNA repair, immunity, and cell growth and proliferation, which have been functionally validated to shorten or prolong lifespan in mice via overexpression, knockdown or knockout experiments [33]. Our results suggest that, while the expression levels of the pro-longevity genes did not significantly differ between these two species, *M. myotis* are likely to achieve longevity by suppressing the expression of some anti-longevity genes. Most remarkably, *IGF1R* and *DGAT1* were down-regulated 23.7-fold and 23.2-fold in long-lived *M. myotis* compared to short-lived *M. molossus*, respectively. *IGF1R* is an insulin receptor which exerts pleiotropic roles in glucose metabolism, cell growth development and survival [34]. However, deregulation of *IGF1R* expression, commonly seen within individuals in their late-life stage, is associated with the occurrence and development of diabetes, inflammation and cancer [34]. It has been reported that *igf1r*-knockout mice live on average 26% longer than their wide-type littermates [35]. In addition, a previous study observed decreased expression of genes involved in insulin/IGF-1 signalling pathways in the liver of naked mole-rat compared with mice [36]. Likewise, the role of *DGAT1* is diverse and its overexpression promotes the development of insulin resistance, obesity and fatty-acid induced inflammation [37, 38]. Deficiency of *DGAT1* protects against the metabolic consequences of aging and extends mean and maximal lifespan in mice [39]. These candidates, together with their expression patterns in long-lived bats, could present promising therapeutic targets to delay aging in humans. However, how *M. myotis* bats naturally regulate and control expression of these genes requires further investigation, particularly on non-coding regions that may underlie the regulatory mechanisms of their longevity adaptations [14].

In summary, comparative genomic and transcriptomic analyses on *M. molossus* and *M. myotis* resulted in the discovery of some genetic signatures that may underlie the exceptional longevity in long-lived bats. We show that, even though most of the genes are under purifying selection, long-lived *M. myotis* bats exhibit a different expression profile of enhanced DNA repair and autophagy pathways, and diminished anti-longevity genes, which likely contributes to their extraordinary long lifespan. In the future, selection tests on multiple pairs of phylogenetically matched long- and short-lived branches are required to address if long-lived bats exhibit convergent signatures of longevity or if each lineage has evolved its own longevity adaptations. In addition, longitudinal studies of bats within different LQs will need to be carried out to identify the age-associated genes and pathways that are differentiated between long- and short-lived bats. As the PSGs and DEGs are suggested on the basis of *in silico* analyses, *in vitro* functional assays are required to confirm their roles in bat longevity.

## MATERIALS AND METHODS

### Genome-wide analysis of *Ka/Ks* substitution rates

To assess the selective pressure acting on protein-coding genes, we analysed genome-wide alignments of 12,467 single-copy orthologs between *M. myotis* and *M. molossus.* These alignments were obtained from the Bat1K project [12, 20]. For each pair of orthologs, we calculated the ratio of nonsynonymous (*Ka*) and synonymous (*Ks*) substitution rates using KaKs_Calculator (v2.0) [40] with the Model Averaging (MA) method. Model averaging weights each candidate model (7 in total) and engages more than one model to estimate average parameters across models. Fisher’s exact test was performed to determine the significance of positive selection. The genes with *Ka/Ks* >1 and adjusted *P*-value (FDR) < 0.05 were considered significant positive selection. We further inspected and filtered the positively selected genes (PSGs) due to short alignment (less than 100 bp) and low sequence coverage (less than 80%).

To ascertain if the PSGs identified are unique to the *M. myotis* and *M. molossus* comparison, we included 4 other bat species, which are equivalent in genome completeness and annotation (*Pipistrellus kuhlii*, *Phyllostomus discolor*, *Rhinolophus ferrumequinum* and *Rousettus aegyptiacus*), and investigated *Ka*/*Ks* ratios of genes via pairwise comparisons (n = 15). The alignments of single-copy orthologs across these six species were obtained from the Bat1K project [12, 20]. The method for *Ka*/*Ks* calculation and the criteria for defining PSGs were the same as stated above.

### Blood sample collection and RNA sequencing

The sampling procedures were undertaken in accordance with the ethical guidelines and permits issued by ‘Arrêté’ by the Préfet du Morbihan and the Ministerio de Ambiente de Panamá. *M*. *myotis* and *M*. *molossus* individuals were captured in Brittany, France and Gamboa, Panama, respectively. The blood sampling procedures were extensively described in [41]. Blood samples were collected in cryotubes (2 ml, Nalgene labware) and were immediately flash frozen in liquid nitrogen. All samples were further preserved at -150°C for long-term storage before total RNA extraction. Whole blood total RNA extraction was carried out following the manufacturer’s protocols (RNAzol^@^ BD kit, catalog no. RB192, Molecular Research Centre, Inc) with modifications as reported in [41]. The samples with RIN scores > 8.0 and total RNA > 2 μg satisfied the criteria for RNA-Seq. In this study, 16 qualified RNA samples (8 each for *M*. *myotis* and *M*. *molossus*) were used for Illumina RNA-Seq library preparation. Prior to extraction, the RNA samples were purified by Turbo DNA-free^TM^ kit (catalog number AM1907, Ambion) to deplete residual DNA, and were further treated with Globin-Zero Gold rRNA Removal kit (Epicentre Illumina) to remove abundant rRNA and globin transcripts. RNA-Seq libraries were prepared using the Tru-Seq Stranded RNA Library Prep kit (Illumina), and further barcoded and sequenced on an Illumina HiSeq2500 sequencer to generate 125-bp paired-end reads. Sample details and statistics of the RNA-Seq libraries are available in Supplementary Table 3.

### Comparative transcriptomic analyses

For each sample, raw reads were trimmed adaptors and low-quality regions (< Q25) using Cutadapt (v.1.14) [42]. The filtered reads were subsequently quantified against the reference genes using Salmon (v0.9) [43] with the following parameters: -*k* 31, -*ISF*. *-k* indicates the length of seed while -*ISF* indicates a library of stranded-specific paired-end reads oriented towards each other. Here, we used 12,467 pairs of single-copy orthologs as references for expression analysis for respective species. The genes which were not expressed in either *M*. *molossus* or *M*. *myotis* were excluded from downstream analysis. Based on gene expression, we computed pairwise Spearman’s correlation coefficients across samples using the R package *cor* (v.3.6.0) [44]. Prior to analysis, raw expression counts were normalized using TMM (Trimmed Mean of M-value) method and further log_2_-transformed. Next, differential gene expression analysis was performed using the R package *DESeq2* [45]. The genes with an FDR < 0.01 were considered differentially expressed genes (DEGs). In addition, we also investigated the distributions of the *Ka*/*Ks* ratios of differentially and non-differentially expressed genes using a Kolmogorov-Smirnov test. We further performed a *χ*^2^ test to ascertain if the PSGs between *M. myotis* and *M. molossus* correlated with the DEGs identified (*P*-value < 0.05 was considered significant).

### Gene expression variation analysis

Differential gene expression analysis measures the difference in mean expression between two groups but does not quantify expression variation within each group. To detect gene expression which may truly represent genetic difference between *M*. *myotis* and *M*. *molossus*, we employed a linear mixed model (LMM) to identify genes with high interspecific but low intraspecific variation. Normalised gene expression values were considered as dependent variables, whereas ‘species’ and ‘sex’ were considered as explanatory variables and were modelled as random effects. The variance from uncharacterized sources was treated as residual variance. The LMM was implemented using the R package *variancePartition* [46]. We focused on the genes for which at least 80% of their expression variation was explained by ‘species’. This resulted in 2,086 genes that were further categorized into two groups depending on their expression in *M. myotis* and *M. molossus*. Using Metascape [47], function enrichment analyses were performed on the genes that had higher expression levels in *M*. *myotis* and *M*. *molossus*, respectively. The GO terms with FDR < 0.05 were considered significantly enriched.

### Differential expression of biological processes between *M. myotis* and *M. molossus*

To identify the biological processes (GO terms) that were differentially expressed between *M*. *myotis* and *M*. *molossus*, we investigated gene expression at the GO term level. To achieve this, we focused on the 2,086 genes that may truly represent the genetic difference between these two species, and performed a GO enrichment analysis using Metascape [47]. The GO terms with FDR < 0.05 were considered significantly enriched terms. Subsequently, we collected all the genes under each enriched term in *M*. *myotis* and *M*. *molossus*, and compared their expression levels using Wilcoxon signed-rank tests (paired mode, one-tailed test). The significance of tests was adjusted by Benjamini-Hochberg FDR, and the GO terms with FDR < 0.05 were considered differentially expressed between *M*. *myotis* and *M*. *molossus*.

### Expression of anti- and pro-longevity genes in *M*. *myotis* and *M*. *molossus*

To further investigate whether *M*. *myotis* harboured a transcriptomic signature of longevity relative to *M*. *molossus*, we compiled a list of anti-longevity genes (n = 19) and pro-longevity genes (n = 28) from GenAge [33]. These genes have been validated to either shorten (anti-longevity) or extend (pro-longevity) lifespan in mice via overexpression, knockdown or knockout. We tested whether their expression significantly differed in these two species using Wilcoxon signed-rank tests (paired mode; one-tailed test), respectively. Our result showed that expression levels of the anti-longevity genes had significantly lower expression (P < 0.05) in long-lived *M. myotis* than short-lived *M. molossus*. To evaluate the significance of this test, we randomly subsampled 19 genes out of 2,086 genes and performed Wilcoxon signed-rank tests as previously described. This was repeated 1,000 times with the *P*-value of each test collected and their distribution further analysed using the R package HDInterval. The list of anti- and pro-longevity genes investigated in this study and their expression levels in *M. myotis* and *M. molossus* blood can be available in Supplementary Table 4.

## Supplementary Material

Supplementary Table 2

Supplementary Table 1

Supplementary Tables 3 and 4

## References

[1] George JC, Bada J, Zeh J, Scott L, Brown SE, O'Hara T, Suydam R. Age and growth estimates of bowhead whales (Balaena mysticetus) via aspartic acid racemization. Can J Zool. 1999; 77:571–580. 10.1139/z99-015

[2] Austad SN. Methusaleh’s zoo: how nature provides us with clues for extending human health span. J Comp Pathol. 2010 (Suppl 1); 142:S10–21. 10.1016/j.jcpa.2009.10.024 19962715 PMC3535457

[3] Austad SN, Fischer KE. Mammalian aging, metabolism, and ecology: evidence from the bats and marsupials. J Gerontol. 1991; 46:B47–53. 10.1093/geronj/46.2.b47 1997563

[4] Podlutsky AJ, Khritankov AM, Ovodov ND, Austad SN. A new field record for bat longevity. J Gerontol A Biol Sci Med Sci. 2005; 60:1366–68. 10.1093/gerona/60.11.1366 16339320

[5] Foley NM, Hughes GM, Huang Z, Clarke M, Jebb D, Whelan CV, Petit EJ, Touzalin F, Farcy O, Jones G, Ransome RD, Kacprzyk J, O’Connell MJ, et al. Growing old, yet staying young: the role of telomeres in bats’ exceptional longevity. Sci Adv. 2018; 4:eaao0926. 10.1126/sciadv.aao0926 29441358 PMC5810611

[6] Healy K, Guillerme T, Finlay S, Kane A, Kelly SB, McClean D, Kelly DJ, Donohue I, Jackson AL, Cooper N. Ecology and mode-of-life explain lifespan variation in birds and mammals. Proc Biol Sci. 2014; 281:20140298. 10.1098/rspb.2014.0298 24741018 PMC4043093

[7] Wilkinson GS, Adams DM. Recurrent evolution of extreme longevity in bats. Biol Lett. 2019; 15:20180860. 10.1098/rsbl.2018.0860 30966896 PMC6501359

[8] Muntané G, Farré X, Rodríguez JA, Pegueroles C, Hughes DA, de Magalhães JP, Gabaldón T, Navarro A. Biological processes modulating longevity across primates: a phylogenetic genome-phenome analysis. Mol Biol Evol. 2018; 35:1990–2004. 10.1093/molbev/msy105 29788292 PMC6063263

[9] Sahm A, Bens M, Henning Y, Vole C, Groth M, Schwab M, Hoffmann S, Platzer M, Szafranski K, Dammann P. Higher gene expression stability during aging in long-lived giant mole-rats than in short-lived rats. Aging (Albany NY). 2018; 10:3938–56. 10.18632/aging.101683 30557854 PMC6326690

[10] Sahm A, Bens M, Szafranski K, Holtze S, Groth M, Görlach M, Calkhoven C, Müller C, Schwab M, Kraus J, Kestler HA, Cellerino A, Burda H, et al. Long-lived rodents reveal signatures of positive selection in genes associated with lifespan. PLoS Genet. 2018; 14:e1007272. 10.1371/journal.pgen.1007272 29570707 PMC5884551

[11] Toren D, Kulaga A, Jethva M, Rubin E, Snezhkina AV, Kudryavtseva AV, Nowicki D, Tacutu R, Moskalev AA, Fraifeld VE. Gray whale transcriptome reveals longevity adaptations associated with DNA repair and ubiquitination. Aging Cell. 2020. [Epub ahead of print]. 10.1111/acel.13158 32515539 PMC7433004

[12] Jebb D, Huang Z, Pippel M, Hughes GM, Lavrichenko K, Devanna P, Winkler S, Jermiin LS, Skirmuntt EC, Katzourakis A, Burkitt-Gray L, Ray DA, Sullivan KAM, et al Six new reference-quality bat genomes illuminate the molecular basis and evolution of bat adaptations. bioRxiv. 2019; 836874 10.1101/836874

[13] Tian X, Seluanov A, Gorbunova V. Molecular mechanisms determining lifespan in short- and long-lived species. Trends Endocrinol Metab. 2017; 28:722–34. 10.1016/j.tem.2017.07.004 28888702 PMC5679293

[14] Huang Z, Whelan CV, Foley NM, Jebb D, Touzalin F, Petit EJ, Puechmaille SJ, Teeling EC. Longitudinal comparative transcriptomics reveals unique mechanisms underlying extended healthspan in bats. Nat Ecol Evol. 2019; 3:1110–20. 10.1038/s41559-019-0913-3 31182815

[15] Jebb D, Foley NM, Whelan CV, Touzalin F, Puechmaille SJ, Teeling EC. Population level mitogenomics of long-lived bats reveals dynamic heteroplasmy and challenges the free radical theory of ageing. Sci Rep. 2018; 8:13634. 10.1038/s41598-018-31093-2 30206380 PMC6134106

[16] Seim I, Fang X, Xiong Z, Lobanov AV, Huang Z, Ma S, Feng Y, Turanov AA, Zhu Y, Lenz TL, Gerashchenko MV, Fan D, Hee Yim S, et al. Genome analysis reveals insights into physiology and longevity of the brandt’s bat myotis brandtii. Nat Commun. 2013; 4:2212. 10.1038/ncomms3212 23962925 PMC3753542

[17] Zhang G, Cowled C, Shi Z, Huang Z, Bishop-Lilly KA, Fang X, Wynne JW, Xiong Z, Baker ML, Zhao W, Tachedjian M, Zhu Y, Zhou P, et al. Comparative analysis of bat genomes provides insight into the evolution of flight and immunity. Science. 2013; 339:456–60. 10.1126/science.1230835 23258410 PMC8782153

[18] Sun L, Wang H, Wang Z, He S, Chen S, Liao D, Wang L, Yan J, Liu W, Lei X, Wang X. Mixed lineage kinase domain-like protein mediates necrosis signaling downstream of RIP3 kinase. Cell. 2012; 148:213–27. 10.1016/j.cell.2011.11.031 22265413

[19] Li D, Meng L, Xu T, Su Y, Liu X, Zhang Z, Wang X. RIPK1-RIPK3-MLKL-dependent necrosis promotes the aging of mouse male reproductive system. Elife. 2017; 6:e27692. 10.7554/eLife.27692 28807105 PMC5557593

[20] Teeling EC, Vernes SC, Dávalos LM, Ray DA, Gilbert MT, Myers E, and Bat1K Consortium. Bat biology, genomes, and the Bat1K project: to generate chromosome-level genomes for all living bat species. Annu Rev Anim Biosci. 2018; 6:23–46. 10.1146/annurev-animal-022516-022811 29166127

[21] Huang Z, Jebb D, Teeling EC. Blood miRNomes and transcriptomes reveal novel longevity mechanisms in the long-lived bat, myotis myotis. BMC Genomics. 2016; 17:906. 10.1186/s12864-016-3227-8 27832764 PMC5103334

[22] Rapaport F, Khanin R, Liang Y, Pirun M, Krek A, Zumbo P, Mason CE, Socci ND, Betel D. Comprehensive evaluation of differential gene expression analysis methods for RNA-seq data. Genome Biol. 2013; 14:R95. 10.1186/gb-2013-14-9-r95 24020486 PMC4054597

[23] Eller MS, Liao X, Liu S, Hanna K, Bäckvall H, Opresko PL, Bohr VA, Gilchrest BA. A role for WRN in telomere-based DNA damage responses. Proc Natl Acad Sci USA. 2006; 103:15073–78. 10.1073/pnas.0607332103 17015833 PMC1586178

[24] Shell SM, Hawkins EK, Tsai MS, Hlaing AS, Rizzo CJ, Chazin WJ. Xeroderma pigmentosum complementation group C protein (XPC) serves as a general sensor of damaged DNA. DNA Repair (Amst). 2013; 12:947–53. 10.1016/j.dnarep.2013.08.013 24051049 PMC3825762

[25] Vijg J, Suh Y. Genome instability and aging. Annu Rev Physiol. 2013; 75:645–68. 10.1146/annurev-physiol-030212-183715 23398157

[26] López-Otín C, Blasco MA, Partridge L, Serrano M, Kroemer G. The hallmarks of aging. Cell. 2013; 153:1194–217. 10.1016/j.cell.2013.05.039 23746838 PMC3836174

[27] Ma S, Upneja A, Galecki A, Tsai YM, Burant CF, Raskind S, Zhang Q, Zhang ZD, Seluanov A, Gorbunova V, Clish CB, Miller RA, Gladyshev VN. Cell culture-based profiling across mammals reveals DNA repair and metabolism as determinants of species longevity. Elife. 2016; 5:e19130. 10.7554/eLife.19130 27874830 PMC5148604

[28] MacRae SL, Croken MM, Calder RB, Aliper A, Milholland B, White RR, Zhavoronkov A, Gladyshev VN, Seluanov A, Gorbunova V, Zhang ZD, Vijg J. DNA repair in species with extreme lifespan differences. Aging (Albany NY). 2015; 7:1171–84. 10.18632/aging.100866 26729707 PMC4712340

[29] Ryter SW, Cloonan SM, Choi AM. Autophagy: a critical regulator of cellular metabolism and homeostasis. Mol Cells. 2013; 36:7–16. 10.1007/s10059-013-0140-8 23708729 PMC3887921

[30] Leidal AM, Levine B, Debnath J. Autophagy and the cell biology of age-related disease. Nat Cell Biol. 2018; 20:1338–48. 10.1038/s41556-018-0235-8 30482941

[31] Zhao Z, Ni D, Ghozalli I, Pirooz SD, Ma B, Liang C. UVRAG: at the crossroad of autophagy and genomic stability. Autophagy. 2012; 8:1392–93. 10.4161/auto.21035 22885520 PMC3442888

[32] Pride H, Yu Z, Sunchu B, Mochnick J, Coles A, Zhang Y, Buffenstein R, Hornsby PJ, Austad SN, Pérez VI. Long-lived species have improved proteostasis compared to phylogenetically-related shorter-lived species. Biochem Biophys Res Commun. 2015; 457:669–75. 10.1016/j.bbrc.2015.01.046 25615820

[33] Tacutu R, Craig T, Budovsky A, Wuttke D, Lehmann G, Taranukha D, Costa J, Fraifeld VE, de Magalhães JP. Human ageing genomic resources: integrated databases and tools for the biology and genetics of ageing. Nucleic Acids Res. 2013; 41:D1027–33. 10.1093/nar/gks1155 23193293 PMC3531213

[34] Lee WS, Kim J. Insulin-like growth factor-1 signaling in cardiac aging. Biochim Biophys Acta Mol Basis Dis. 2018; 1864:1931–38. 10.1016/j.bbadis.2017.08.029 28847512

[35] Holzenberger M, Dupont J, Ducos B, Leneuve P, Géloën A, Even PC, Cervera P, Le Bouc Y. IGF-1 receptor regulates lifespan and resistance to oxidative stress in mice. Nature. 2003; 421:182–87. 10.1038/nature01298 12483226

[36] Kim EB, Fang X, Fushan AA, Huang Z, Lobanov AV, Han L, Marino SM, Sun X, Turanov AA, Yang P, Yim SH, Zhao X, Kasaikina MV, et al. Genome sequencing reveals insights into physiology and longevity of the naked mole rat. Nature. 2011; 479:223–27. 10.1038/nature10533 21993625 PMC3319411

[37] Koliwad SK, Streeper RS, Monetti M, Cornelissen I, Chan L, Terayama K, Naylor S, Rao M, Hubbard B, Farese RV Jr. DGAT1-dependent triacylglycerol storage by macrophages protects mice from diet-induced insulin resistance and inflammation. J Clin Invest. 2010; 120:756–67. 10.1172/JCI36066 20124729 PMC2827941

[38] Smith SJ, Cases S, Jensen DR, Chen HC, Sande E, Tow B, Sanan DA, Raber J, Eckel RH, Farese RV Jr. Obesity resistance and multiple mechanisms of triglyceride synthesis in mice lacking dgat. Nat Genet. 2000; 25:87–90. 10.1038/75651 10802663

[39] Streeper RS, Grueter CA, Salomonis N, Cases S, Levin MC, Koliwad SK, Zhou P, Hirschey MD, Verdin E, Farese RV Jr. Deficiency of the lipid synthesis enzyme, DGAT1, extends longevity in mice. Aging (Albany NY). 2012; 4:13–27. 10.18632/aging.100424 22291164 PMC3292902

[40] Wang D, Zhang Y, Zhang Z, Zhu J, Yu J. KaKs_Calculator 2.0: a toolkit incorporating gamma-series methods and sliding window strategies. Genomics Proteomics Bioinformatics. 2010; 8:77–80. 10.1016/S1672-0229(10)60008-3 20451164 PMC5054116

[41] Huang Z, Gallot A, Lao NT, Puechmaille SJ, Foley NM, Jebb D, Bekaert M, Teeling EC. A nonlethal sampling method to obtain, generate and assemble whole blood transcriptomes from small, wild mammals. Mol Ecol Resour. 2016; 16:150–62. 10.1111/1755-0998.12447 26186236

[42] Martin M. Cutadapt removes adapter sequences from high-throughput sequencing reads. EMBnet J. 2011; 17:pp. 10–12. 10.14806/ej.17.1.200

[43] Patro R, Duggal G, Love MI, Irizarry RA, Kingsford C. Salmon provides fast and bias-aware quantification of transcript expression. Nat Methods. 2017; 14:417–19. 10.1038/nmeth.4197 28263959 PMC5600148

[44] Team RC. (2014). R: A language and environment for statistical computing. R Foundation for Statistical Computing, Vienna, Austria, 2012 ISBN 3-900051-07-0).

[45] Love MI, Huber W, Anders S. Moderated estimation of fold change and dispersion for RNA-seq data with DESeq2. Genome Biol. 2014; 15:550. 10.1186/s13059-014-0550-8 25516281 PMC4302049

[46] Hoffman GE, Schadt EE. variancePartition: interpreting drivers of variation in complex gene expression studies. BMC Bioinformatics. 2016; 17:483. 10.1186/s12859-016-1323-z 27884101 PMC5123296

[47] Zhou Y, Zhou B, Pache L, Chang M, Khodabakhshi AH, Tanaseichuk O, Benner C, Chanda SK. Metascape provides a biologist-oriented resource for the analysis of systems-level datasets. Nat Commun. 2019; 10:1523. 10.1038/s41467-019-09234-6 30944313 PMC6447622

