# Supplementary material for: Genetic variation between long-lived versus short-lived bats illuminates the molecular signatures of longevity
Source: Aging (Albany NY). 2020 Aug 31;12(16):15962–77. doi: 10.18632/aging.103725 (PMC7485743; doi:10.18632/aging.103725)
Supplement: Supplementary Table 1 [file aging-12-103725-s003..pdf]

SUPPLEMENTARY TABLE

Supplementary Table 1. Positive selected genes (PSGs) identified among 6 bat species through pairwise comparisons.

Ensembl Transcript ID	Gene Symbol	Ka	Ks	Ka/Ks	FDR
Molossus molossus versus *Pipistrellus kuhlii*					
ENST00000537690	*CCDC175*	0.3273	0.2503	1.31	0.0036
Molossus molossus versus *Phyllostomus discolor*					
ENST00000393316	*BCL2L15*	0.314	0.1867	1.682	0.0156
ENST00000618484	*BPIFA1*	0.5722	0.3442	1.662	0.0001
Molossus molossus versus *Rhinolophus ferrumequinum*					
ENST00000378186	*MS4A13*	0.33	0.1873	1.762	0.0274
ENST00000380232	*IFNB1*	0.4029	0.259	1.556	0.0424
ENST00000368051	*CD84*	0.2354	0.1565	1.504	0.03
ENST00000537690	*CCDC175*	0.2651	0.2173	1.22	0.0436
Molossus molossus versus *Rousettus aegyptiacus*					
ENST00000369102	*C1orf54*	0.2231	0.0985	2.264	0.0111
ENST00000537690	*CCDC175*	0.268	0.2173	1.233	0.0336

Ensembl Transcript ID	Gene Symbol	Ka	Ks	Ka/Ks	FDR
Myotis myotis versus *Phyllostomus discolor*					
NA					
Myotis myotis versus *Pipistrellus kuhlii*					
ENST00000391930	*IL20*	0.1981	0.0841	2.357	0.0061
ENST00000378186	*MS4A13*	0.1849	0.0831	2.226	0.0131
ENST00000546561	*TSPAN8*	0.2668	0.1396	1.911	0.0016
ENST00000389019	*SLCO6A1*	0.1507	0.0912	1.653	0.0159
ENST00000537690	*CCDC175*	0.1775	0.115	1.543	0.0013
ENST00000273352	*ADGRG7*	0.1874	0.1474	1.272	0.0477
Myotis myotis versus *Rhinolophus ferrumequinum*					
ENST00000372670	*WFDC6*	0.2698	0.1064	2.535	0.0304
ENST00000370350	*FATE1*	0.3787	0.2216	1.79	0.0074
ENST00000537690	*CCDC175*	0.321	0.2407	1.334	0.0014
Myotis myotis versus *Rousettus aegyptiacus*					
ENST00000445202	*PATE3*	0.2811	0.1345	2.091	0.0358
ENST00000381627	*RLN2*	0.3601	0.1987	1.812	0.0205
ENST00000370350	*FATE1*	0.3579	0.2039	1.755	0.0037
ENST00000537690	*CCDC175*	0.3025	0.2511	1.205	0.0439

Ensembl Transcript ID	Gene Symbol	Ka	Ks	Ka/Ks	FDR
Phyllostomus discolor versus *Pipistrellus kuhlii*					
ENST00000546561	*TSPAN8*	0.3542	0.2529	1.4	0.0373
Phyllostomus discolor versus *Rhinolophus ferrumequinum*					
ENST00000296739	*SPZ1*	0.2467	0.1614	1.528	0.0072
Phyllostomus discolor versus *Rousettus aegyptiacus*					
ENST00000371431	*AKAP19*	0.2644	0.1427	1.853	0.037
Pipistrellus kuhlii versus *Rhinolophus ferrumequinum*					
ENST00000378186	*MS4A13*	0.3606	0.1745	2.066	0.0023
ENST00000370250	*FATE1*	0.419	0.2327	1.801	0.0053
ENST00000537690	*CCDC175*	0.3427	0.2656	1.29	0.0047
Pipistrellus kuhlii versus *Rousettus aegyptiacus*					
NA					
Rhinolophus ferrumequinum versus *Rhinolophus ferrumequinum*					
ENST00000382208	*DEFB135*	0.3642	0.1551	2.349	0.0129

Genes that exhibited *Ka/Ks* > 1 and FDR < 0.05 (Fisher exact test) were considered positively selected genes.
