# Supplementary material for: Genetic variation between long-lived versus short-lived bats illuminates the molecular signatures of longevity
Source: Aging (Albany NY). 2020 Aug 31;12(16):15962–77. doi: 10.18632/aging.103725 (PMC7485743; doi:10.18632/aging.103725)
Supplement: Supplementary Tables 3 and 4 [file aging-12-103725-s002..pdf]

SUPPLEMENTARY TABLES

Supplementary Table 3. Information of *M. myotis* and *M. molossus* RNA-Seq samples.

Species	Sample ID	Age	Sex	Raw reads	Clean reads
M. molossus	AHKX-1	Adult	Female	32,087,001	29,122,203
	AHKX-2	Adult	Male	39,138,864	35,776,979
	AHKX-3	Adult	Female	34,994,963	32,206,385
	AHKX-4	Adult	Male	42,599,132	38,612,182
	AHKX-5	Adult	Female	44,567,553	40,710,399
	AHKX-6	Adult	Female	39,533,956	36,065,884
	AHKX-7	Adult	Male	41,315,334	38,443,201
	AHKX-8	Adult	Female	41,544,292	38,563,116
M. myotis	AJCC-7	Adult	Female	69,608,563	63,479,697
	AJCC-9	Adult	Female	56,347,739	50,896,083
	AJCC-11	Adult	Female	57,600,266	54,034,263
	AJCC-13	Adult	Female	75,867,273	70,421,670
	AJCC-17	Adult	Female	61,537,380	55,193,047
	AJCC-18	Adult	Female	66,857,830	60,145,110
	AJCC-22	Adult	Female	60,840,476	55,478,542
	AJCC-27	Adult	Female	69,521,460	63,916,035

Supplementary Table 4. The expression of anti- and pro-longevity genes in *M. myotis* and *M. molossus*.

TMM normalized expression of anti-longevity genes			
**Gene**	**Description**	***M.myotis***	***M. molossus***
INSR	Insulin receptor	2792.163	5929.826
GSTA4	Glutathione S-transferase Alpha 4	89.007	1284.609
TERF2	Telomeric repeat binding factor 2	251.694	31.528
COQ7	Cozenzyme Q7, Hydroxylase	57.091	328.937
EIF5A2	Eukaryotic translation initiation factor 5A2	27.979	21.402
EEF1E1	Eukaryotic translation elongation factor 1 Epsilon 1	20.796	5.661
EPS8	Epidermal growth factor receptor pathway substrate 8	139.443	1.307
CDKN1A	Cyclin dependent kinase inhibitor 1A	10.976	43.224
MTOR	Mechanistic target of rapamycin kinase	279.099	285.992
BAX	BCL2 associated X, apoptosis regulator	14.122	22.982
PARP1	Poly(ADP-ribose) polymerase 1	74.309	233.679
SHC1	SHC adaptor protein 1	25.109	57.983
KCNA3	Potassium voltage-gated channel subfamily A member 3	15.122	36.579
IGF1R	Insulin like growth factor 1 receptor	22.789	540.124
MYC	MYC proto-oncogene, BHLH transcription factor	12.103	8.237
ADCY5	Adenylate cyclase 5	19.26	0
DGAT1	Diacylglycerol O-acyltransferase 1	3.479	80.706
MIF	Macrophage migration inhibitory factor	0	5.14
GHR	Growth hormone receptor	3.1	0

TMM normalized expression of pro-longevity genes			
**Gene**	**Description**	***M. myotis***	***M. molossus***
RICTOR	RPTOR independent companion of MTOR complex 2	12869.042	595.476
ARHGAP1	Rho GTPase activating protein 1	43.247	38.333
BUB1B	BUB1 mitotic checkpoint serine/threonine kinase	7222.727	4630.683
PTEN	Phosphatase and tensin homolog	1830.856	0
ZMPSTE24	Zinc metallopeptidase STE24	1628.613	393.672
UCP2	Uncoupling protien 2	577.02	2683.561
TPP2	Tripeptidyl peptidase 2	2404.549	955.757
PPM1D	Protein Phosphatase, Mg2+/Mn2+ dependent 1D	1184.73	7689.797
CDK7	Cyclin dependent kinase 7	552.9	146.54
CISD2	CDGSH iron sulfur domain 2	150.344	216.23
SQSTM1	Sequestosome 1	456.844	361.879
PRDX1	Peroxiredoxin 1	184.507	58.715
SOD2	Superoxide dismutase 2	103.747	353.785
STUB1	STIP1 homology and U-box containing protein 1	166.509	44.34
GRN	Granulin precursor	261.758	33.561
SIRT1	Sirtuin 1	372.305	509.23
GSK3A	Glycogen synthase kinase 3 Alpha	148.867	300.306
ATM	ATM serine/threonine kinase	1244.425	5006.536
CLOCK	Clock circadian regulator	203.001	22.427
XRCC5	X-ray repair cross complementing 5	137.304	190.863
CAT	Catalase	0	10.697
FOXM1	Forkhead box M1	107.912	225.901
RAE1	Ribonucleic acid export 1	36.031	293.434
MSRA	Methionine sulfoxide reductase A	11.906	4.044
TOP3B	DNA topoisomerase III Beta	50.827	61.747
ARNTL	Aryl hydrocarbon receptor nuclear translocator like	28.035	13.074
NUDT1	Nudix hydrolase 1	0	57.421
SIRT6	Surtuin 6	0.381	5.36

Note: for each species, gene expression value is represented by the median of TMM normalized expression across all samples (n=8)
Gene expression values were normalized using Trimmed Mean of M-value (TMM) method.
